# Methylation silencing of TGF-β receptor type II is involved in malignant transformation of esophageal squamous cell carcinoma

**DOI:** 10.1186/s13148-020-0819-6

**Published:** 2020-02-11

**Authors:** Yarui Ma, Siyuan He, Aiai Gao, Ying Zhang, Qing Zhu, Pei Wang, Beibei Yang, Huihui Yin, Yifei Li, Jinge Song, Pinli Yue, Mo Li, Dandan Zhang, Yun Liu, Xiaobing Wang, Mingzhou Guo, Yuchen Jiao

**Affiliations:** 1 State Key Lab of Molecular Oncology, National Cancer Center/National Clinical Research Center for Cancer/Cancer Hospital, Chinese Academy of Medical Sciences and Peking Union Medical College, Research Building, No.17 Panjiayuan Nanli, Chaoyang District, Beijing, 100021 China; 2 Department of Gastroenterology & Hepatology, Chinese PLA General Hospital, Research Building, No.28 Fuxing Road, Haidian District, Beijing, 100853 China; 3 MOE Key Laboratory of Metabolism and Molecular Medicine, Department of Biochemistry and Molecular Biology, School of Basic Medical Sciences and Zhongshan Hospital, Fudan University, Shanghai, 200032 China

**Keywords:** Esophageal squamous cell carcinoma, Whole genome bisulfite sequencing, TGFBR2, Methylation changes, Cancer diagnosis, Treatment

## Abstract

**Background:**

Although massive studies have been conducted to investigate the mechanisms of esophageal squamous cell carcinoma (ESCC) carcinogenesis, the understanding of molecular alterations during the malignant transformation of epithelial dysplasia is still lacking, especially regarding epigenetic changes.

**Results:**

To better characterize the methylation changes during the malignant transformation of epithelial dysplasia, a whole-genome bisulfite sequencing analysis was performed on a series of tumor, dysplastic, and non-neoplastic epithelial tissue samples from esophageal squamous cell carcinoma (ESCC) patients. Promoter hypermethylation in TGF-β receptor type II (TGFBR2), an important mediator of TGF-β signaling, was identified. Further, we evaluated the methylation and expression of TGFBR2 in tumor samples through The Cancer Genome Atlas multiplatform data as well as immunohistochemistry. Moreover, treatment of ESCC cell lines with5-Aza-2′-deoxycytidine, a DNA methyltransferase inhibitor, reactivated the expression of TGFBR2. The lentiviral mediating the overexpression of TGFBR2 inhibited the proliferation of ESCC cell line by inducing cell cycle G2/M arrest. Furthermore, the overexpression of TGFBR2 inhibited the tumor growth obviously in vivo.

**Conclusions:**

The characterization of methylation silencing of TGFBR2 in ESCC will enable us to further explore whether this epigenetic change could be considered as a predictor of malignant transformation in esophageal epithelial dysplasia and whether use of a TGFBR2 agonist may lead to a new therapeutic strategy in patients with ESCC.

## Background

Esophageal cancer (EC) is one of the common malignant tumors in China [1, 2]. The incidence of EC in China accounts for about 50% of new EC patients occurring worldwide [3, 4]. Notably, esophageal squamous cell carcinoma (ESCC) represents the predominant histological type with a 90% prevalence in China [2, 5]. The standard treatments include surgery and chemotherapy, but due to the absence of obvious symptoms in early stage of ESCC, patients are frequently diagnosed only after reaching an advanced stage [6, 7]. The 5-year overall survival rate following comprehensive treatment for advanced patients is approximately 25–30%, and these treatments are often accompanied by highly undesirable side effects. However, surgical treatment at early stages for ESCC may increase the 5-year survival rate to 70%. Therefore, there is a need for further in-depth study of disease mechanism and the development of new treatment strategies.

It is widely recognized that the carcinogenesis of ESCC is a multistep process, which progresses from dysplasia and involves multiple genetic changes [8]. Epigenetic changes including DNA methylation play a critical character in the management of gene expression patterns and are independent of mutations in the DNA sequence. Methylation of CpG islands in promoter regions frequently contributing to gene transcriptional silencing may serve as an important mechanism to inactivate tumor suppressor genes in cancer [9–11]. Moreover, promoter methylation occurs early in the development of cancer. Thus, the identification of methylation changes in tumor suppressor genes is of tremendous importance since it could contribute to early detection and new drug development for ESCC patients.

Although tumor methylomes have been extensively characterized, conventional methods, such as methylation-specific PCR (MSP), or enrichment-based approaches, such as methylated DNA immunoprecipitation sequencing (MeDIP-Seq), have been mostly used to study methylation changes in cancers including ESCC [12–16]. However, a more comprehensive landscape of the ESCC methylome is still lacking. In addition, little is known about the development of dysplasia into ESCC. Recent advances in high throughput sequencing enable mapping of DNA methylation at single-base resolution (whole genome bisulfite sequencing; WGBS), which would also help to comprehensively characterize changes in DNA methylation. As such, it is necessary to re-evaluate DNA methylation changes during the development of ESCC by comparing esophageal tumors with corresponding normal as well as dysplastic tissues.

In this study, the transforming growth factor-β (TGF-β) receptor type II gene (*TGFBR2*), a key mediator of TGF-β signaling which has been implicated in ESCC carcinogenesis, was identified as a putative tumor suppressor in ESCC based on WGBS of paired and unpaired ESCC tissues. We further investigated *TGFBR2* methylation status and expression level both in ESCC tissues and cell lines, and determined the relationship between *TGFBR2* and ESCC. This study provides significant insight into the epigenetic regulation in ESCC associated with *TGFBR2* which could be a potential molecular target in the ESCC diagnosis and treatment.

## Results

### Methylation landscape of esophageal squamous dysplasia and ESCC

To better characterize the methylation profiles of ESCC and precursor lesions, and the relationship between them, we performed whole-genome bisulfite sequencing (WGBS) on ESCC (*n* = 3), dysplastic (*n* = 7), and non-neoplastic epithelial tissue samples (*n* = 6) from ESCC patients. From two of these patients, we obtained matched non-neoplastic, dysplastic, and tumor samples. On average, the alignment rate of sequencing read mapping to the reference genome was 87.8%. The average coverage of all libraries was 6.83-fold. To evaluate the bisulfite conversion rate, unmethylated lambda DNA was spiked in as the control during library construction. The average bisulfite conversion (unmethylated cytosine to uracil) rate was considerably high (99.2%). Two of the samples were of insufficient quality and therefore did not undergo further analysis.

**Fig. 1:**
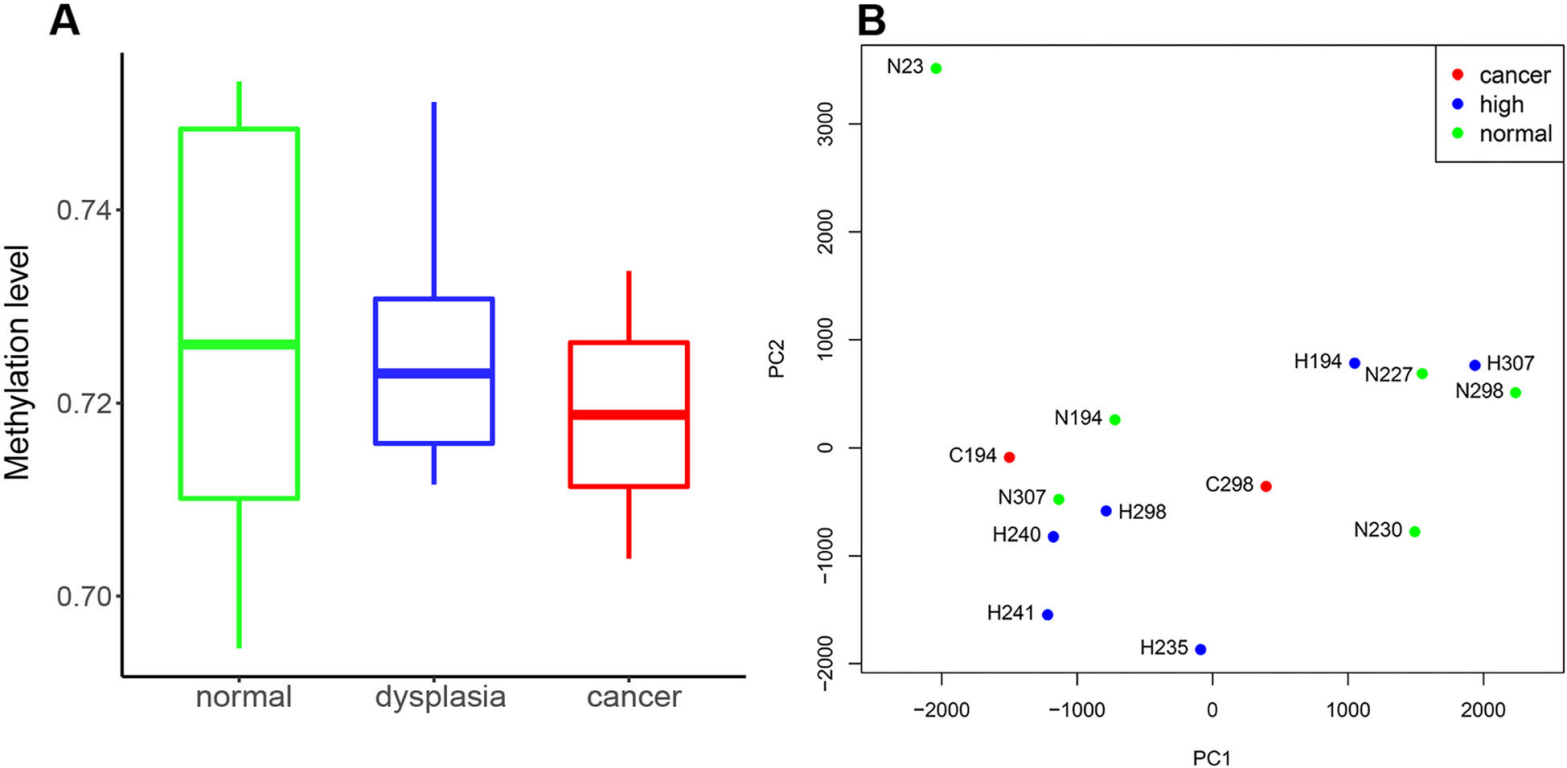
Whole genome methylation profiling of ESCC and esophageal dysplasia samples. **a** Genome-wide methylation level of ESCC, dysplasia, and non-neoplastic samples. **b** Principal component analysis of whole genome bisulfite sequencing data

WGBS data revealed a bimodal distribution of methylation in these samples. However, the genome-wide methylation levels of non-neoplastic, dysplastic, and ESCC samples did not differ significantly. Only a trend toward a decrease in the methylation levels from non-neoplastic to cancer tissue samples emerged from these data (Fig. 1a). In addition, using the principal component analysis, we observed a relatively high degree of epigenetic heterogeneity between non-neoplastic, dysplastic, and cancer samples in each patient (Fig. 1b). These results support the previous finding, based on the genomic analysis of ESCC, that significant heterogeneity exists between matched dysplastic and ESCC samples in patients [17].

### Characterization of differentially methylated regions in dysplasia and ESCC

**Fig. 2:**
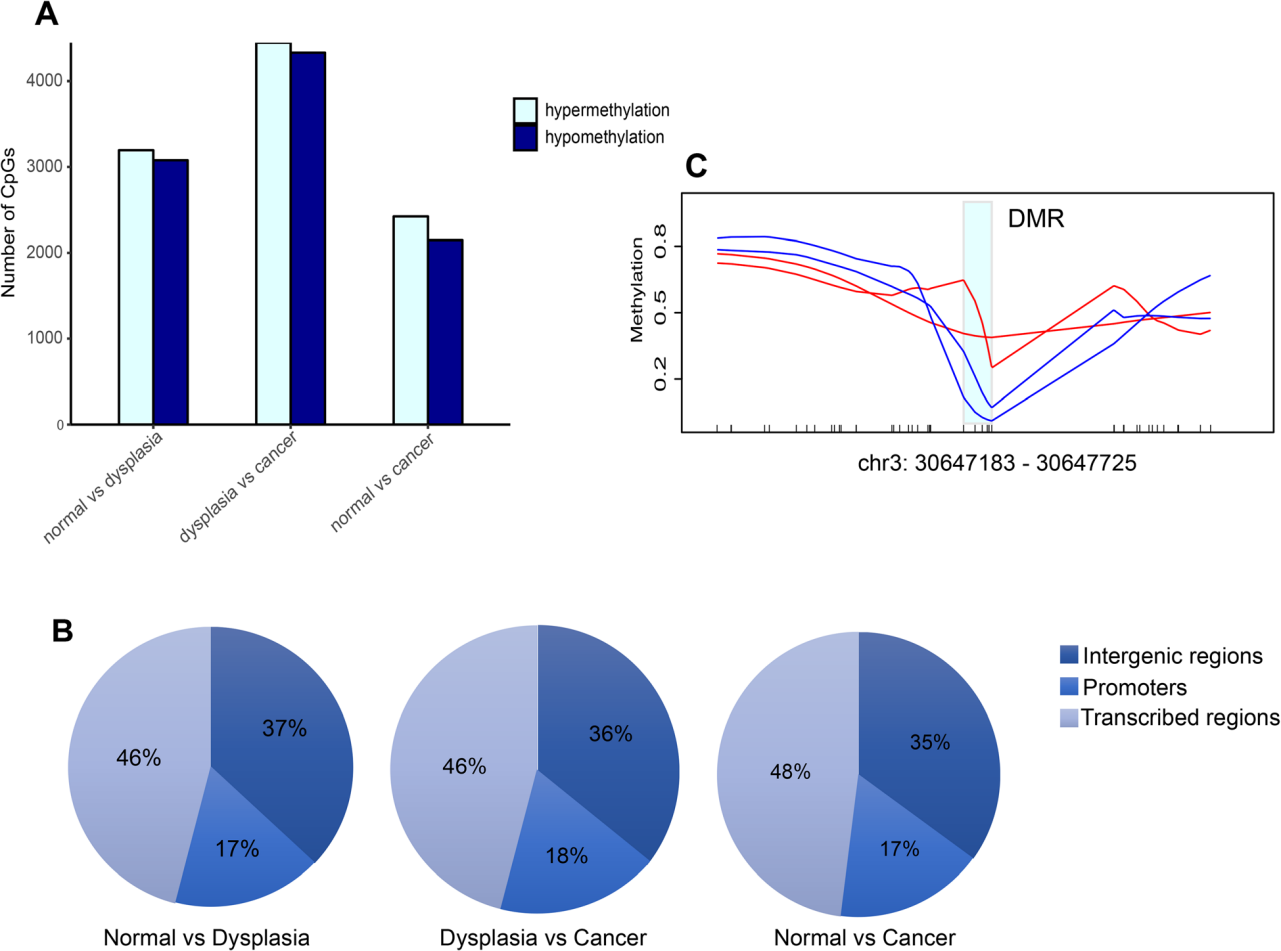
Differential DNA methylation in esophageal dysplasia and ESCC. **a** Distribution of hypermethylated and hypomethylated CpG sites between different stages. **b** Proportion of differentially methylated regions in transcribed regions, intergenic regions, and promoters. **c** Methylation difference in TGFBR2 promoter between dysplastic and tumor stages in two paired samples

To investigate whether DNA methylation variations are associated with alterations in gene expression in the development of ESCC, we identified differentially methylated regions (DMRs) between the different stages. In total, we identified 969 differentially methylated regions between non-neoplastic and tumor samples, 1293 DMRs between non-neoplastic and dysplastic samples, and 1838 DMRs between dysplastic and tumor samples. There were no obvious differences in the number between hypermethylated and hypomethylated CpG sites in these comparisons (Fig. 2a). In addition, almost half of these DMRs were located in transcribed regions (including transcriptional start sites to transcriptional end sites) rather than in intergenic or promoter regions (Fig. 2b). In some genes, including *LHFPL6* and *ABL2*, promoter methylation from the non-neoplastic to the dysplastic stage had decreased. This result suggested that in these genes, promoter hypomethylation likely occurred very early in ESCC development and might therefore serve as potential biomarkers for the diagnosis of esophageal dysplasia. We did not observe promoter hypermethylation in certain tumor suppressor genes such as *CDKN2A* [18], *TFF1* [14], and *CDH1* [19] as previously reported in other cohorts. However, we found promoter hypermethylation in the putative tumor suppressor gene *TGFBR2* during the transition from dysplasia to ESCC. We observed similar results when comparing methylation levels in two paired samples (Fig. 2c). The *TGFBR2* promoter exhibited hypermethylation not only in the transition from dysplasia to cancer, but also from normal epithelium to cancer. Notably, previous studies have reported mutations in *TGFBR2* in ESCC, but at a comparatively low mutation rate [20, 21].

### TGFBR2 mRNA and protein are decreased in primary tumor samples relative to normal tissue

**Fig. 3:**
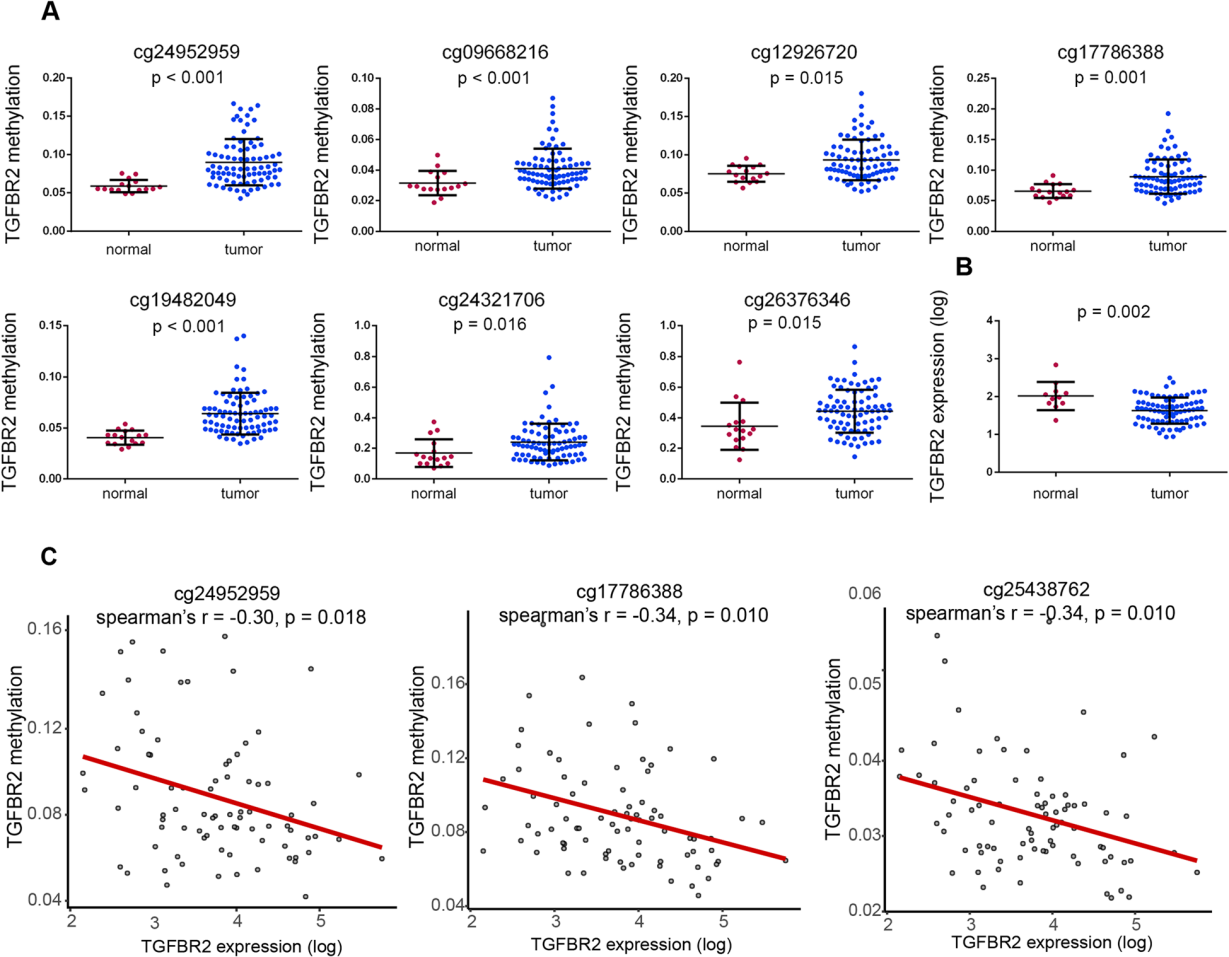
TGFBR2 is hypermethylated and downregulated in TCGA ESCC dataset. **a** DNA methylation comparison of TGFBR2 promoter-associated CpG sites in normal and tumor samples. **b** Expression levels of TGFBR2 in normal and ESCC samples. **c** Correlations of promoter methylation and expression for TGFBR2

**Fig. 4:**
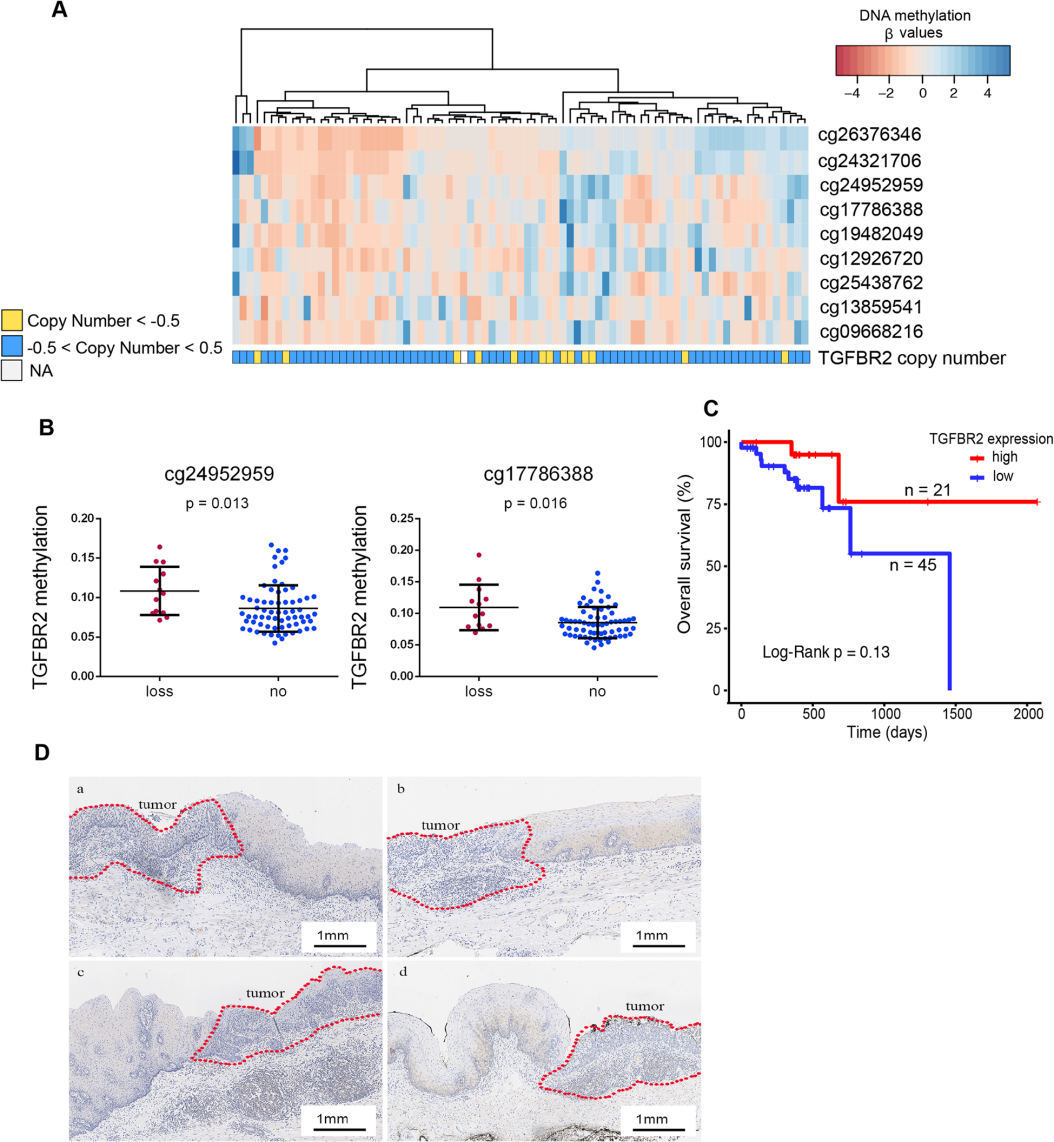
TGFBR2 expression and its relationship with copy number and patients’ outcome. **a**, **b** Correlation of TGFBR2 copy number and promoter methylation. **c** Kaplan-Meier curves of overall survival according to TGFBR2 expression level. **d** IHC performed on sections from ESCC and adjacent tissues with TGFBR2 antibody

To examine the association between *TGFBR2* methylation and expression levels, we explored the multiplatform *TGFBR2* profiles, including methylation, RNA-seq, and copy number data for 81 ESCC samples and 16 adjacent normal tissue samples from the TCGA dataset. CpG sites of the *TGFBR2* promoter were significantly hypermethylated in tumor samples compared to normal samples (Fig. 3a). Based on the RNA-seq data, *TGFBR2* was downregulated in tumor relative to normal tissue samples (Fig. 3b). To investigate the influence of DNA methylation on the gene expression, we calculated the Spearman rank correlation coefficient between methylation of each *TGFBR2* promoter CpG site and the expression level. All three CpG sites were significantly negatively correlated with *TGFBR2* expression (Fig. 3c). We also examined the relationship between *TGFBR2* copy number and methylation levels. Interestingly, samples with copy number loss had significantly higher methylation levels in two CpG sites (Fig. 4a, b), which implicated the loss of tumor suppression gene function in accord with Knudson’s two-hit theory. Finally, we examined whether *TGFBR2* expression levels were associated with clinical outcome. Although not statistically significant, patients with high *TGFBR2* expression had a relatively favor prognosis compared to those with low expression levels (expression threshold 63.5; Fig. 4c).

We validated these results by performing IHC on 66 samples from our own cohort. In 34 of these samples, the level of TGFBR2 protein was downregulated significantly in tumor tissues compared to dysplastic and normal tissue samples (Fig. 4d).

### Promoter hypermethylation is associated with TGFBR2 transcriptional silencing in ESCC cell lines

**Fig. 5:**
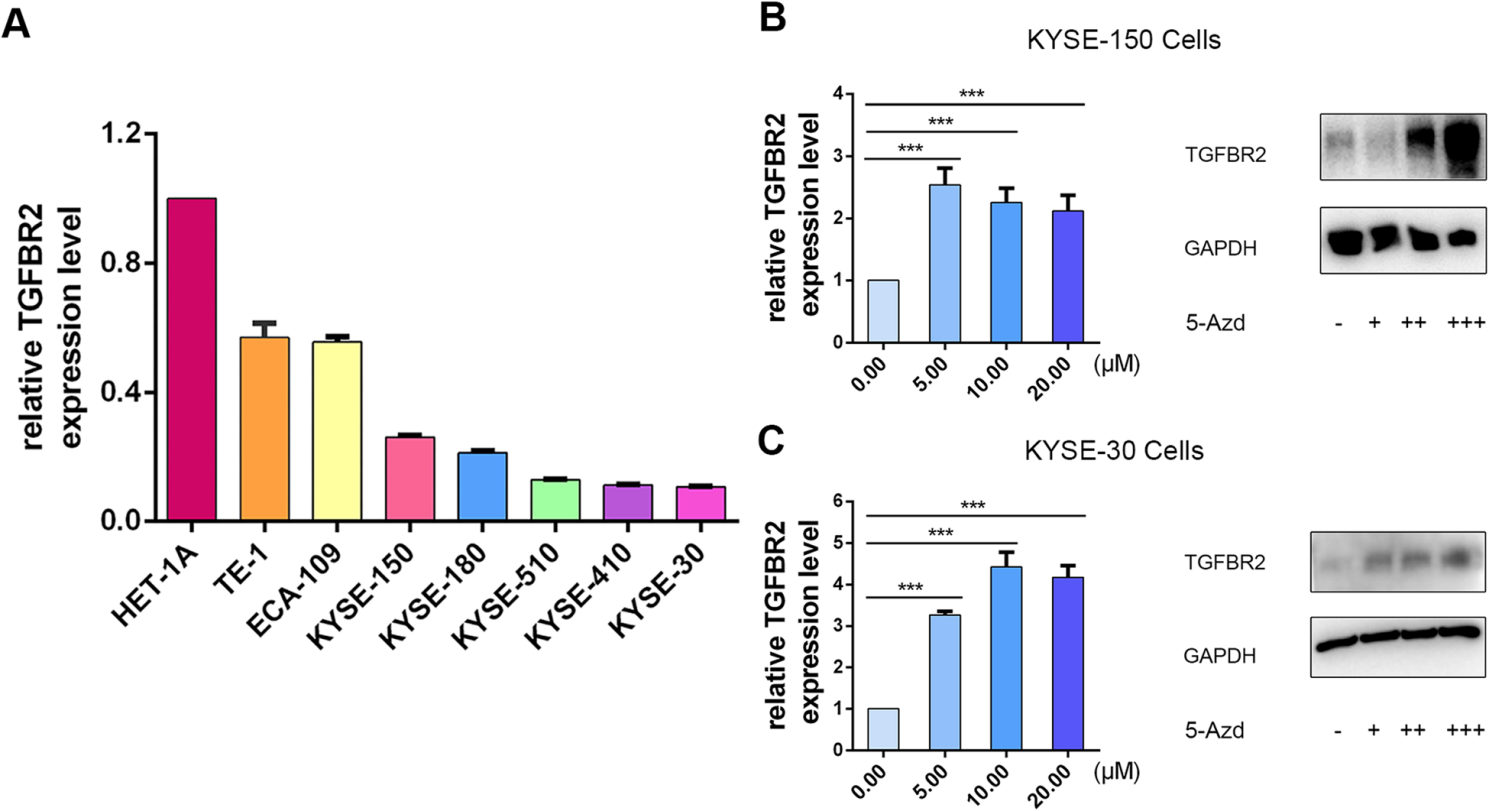
Treatment of ESCC cells in culture increases the expression of TGFBR2. **a** RT-qPCR to detect levels of TGFBR2 mRNA in Het-1A and ESCC cell lines using GAPDH as a control gene. **b** RT-qPCR and western blot analysis performed on RNA and protein isolated from KYSE-150 cells exposed to increasing concentrations of 5-Aza-2′-deoxycytidine (mean ± SD. *P* < 0.001). **c** RT-qPCR and western blot analysis performed on RNA and protein isolated from KYSE-30 cells exposed to increasing concentrations of 5-Aza-2′-deoxycytidine (mean ± SD. *P* < 0.001)

Using RT-qPCR, we obtained that *TGFBR2* mRNA levels were also downregulated in several ESCC cell lines (HET-1A, TE-1, ECA-109, KYSE-150, KYSE-180, KYSE-510, KYSE-410, KYSE-30) dramatically compared to an immortalized esophageal epithelial cell line, Het-1A (Fig. 5a, Additional file 1: Figure S1; *P* < 0.05). To further explore the association between gene expression and the promoter methylation status of *TGFBR2*, we examined the expression of *TGFBR2* in KYSE-150 cells exposed to the treatment with 5-Aza-2′-deoxycytidine. We treated KYSE-150 cells with different dosages of the DNA methyltransferase inhibitor 5-Aza-2′-deoxycytidine. The results of RT-qPCR illustrated that the *TGFBR2* expression was significantly upregulated in cells exposed to increasing concentrations of 5-Aza-2′-deoxycytidine (Fig. 5b). Western blot analysis corroborated these results (Fig. 5b). The same results were observed in KYSE-30 cells (Fig. 5c). In summary, these data demonstrated that the methylation of promoter mediated transcriptional silencing of *TGFBR2* in ESCC cell lines.

### TGFBR2 induces ESCC cell cycle arrest but not cell apoptosis

**Fig. 6:**
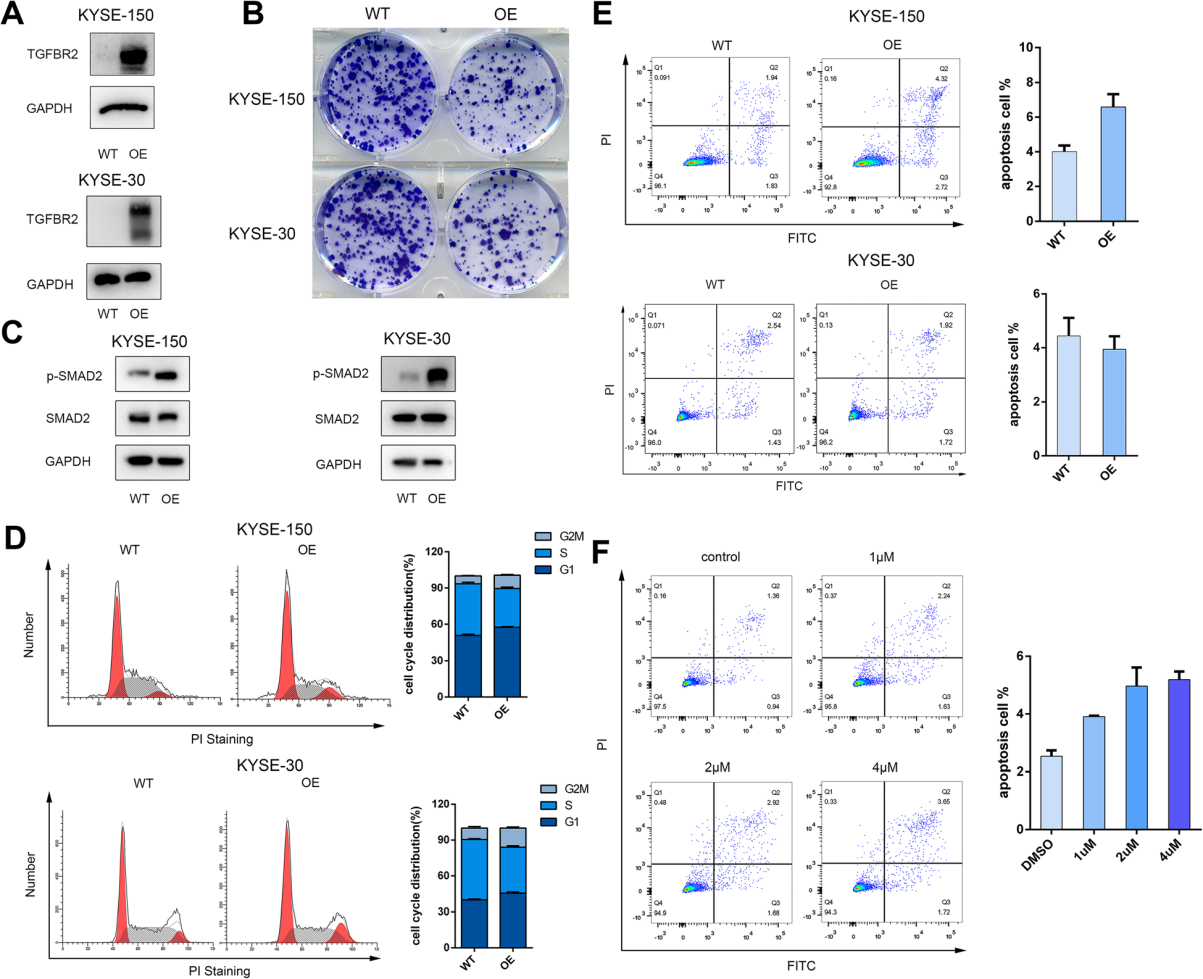
TGFBR2 overexpression induces ESCC cell cycle arrest but not cell apoptosis. **a** Western blot analysis of wild-type (WT) cells and lentiviral mediating the overexpression of TGFBR2 (OE) cells in KYSE-150 and KYSE-30 cell lines. **b** The morphology of WT and OE cells in colony formation assay. **c** The expression level of phospho-SMAD2 and SMAD2 in WT and OE cells. **d** Cell cycle distribution in WT and OE cells. Graphic representation of results from cell cycle analysis in WT and OE cells. **e** Annexin V staining of parental WT and KO cells to detect apoptosis using flow cytometry. Graphic representation of the percentage of apoptotic cells in parental versus OE cells. **f** Annexin V staining of KYSE-150 cells exposed to increasing concentrations of 5-Aza-2′-deoxycytidine detected with flow cytometry. Graphic representation of the percentage of apoptotic cells with increasing 5-Aza-2′-deoxycytidine concentration

To validate whether the expression of *TGFBR2* could prevent the tumor progression, we constructed the TGFBR2 overexpression tumor cells in KYSE-150 and KYSE-30 (Fig. 6a). The expression of phospho-SMAD2 was dramatically reactivated in the TGFBR2 overexpression cells which suggested the TGFβ signaling is restored (Fig. 6b). Overexpression of TGFBR2 suppressed the growth of tumor cells significantly through colony formation assays (Fig. 6c). Consistently, the TGFBR2 overexpression cells induced cell cycle G2/M arrest relative to the wild-type cells as determined through flow cytometry analysis of propidium staining (Fig. 6c). However, 5-Aza-2′-deoxycytidine treatment or TGFBR2 overexpression did not increase the apoptosis rate obviously (Fig. 6d). Collectively, these results suggested that TGFBR2 inhibits the growth of cells by inducing the cell cycle G2/M delayed, but not due to causing cell apoptosis.

### TGFBR2 suppresses ESCC growth in vivo

**Fig. 7:**
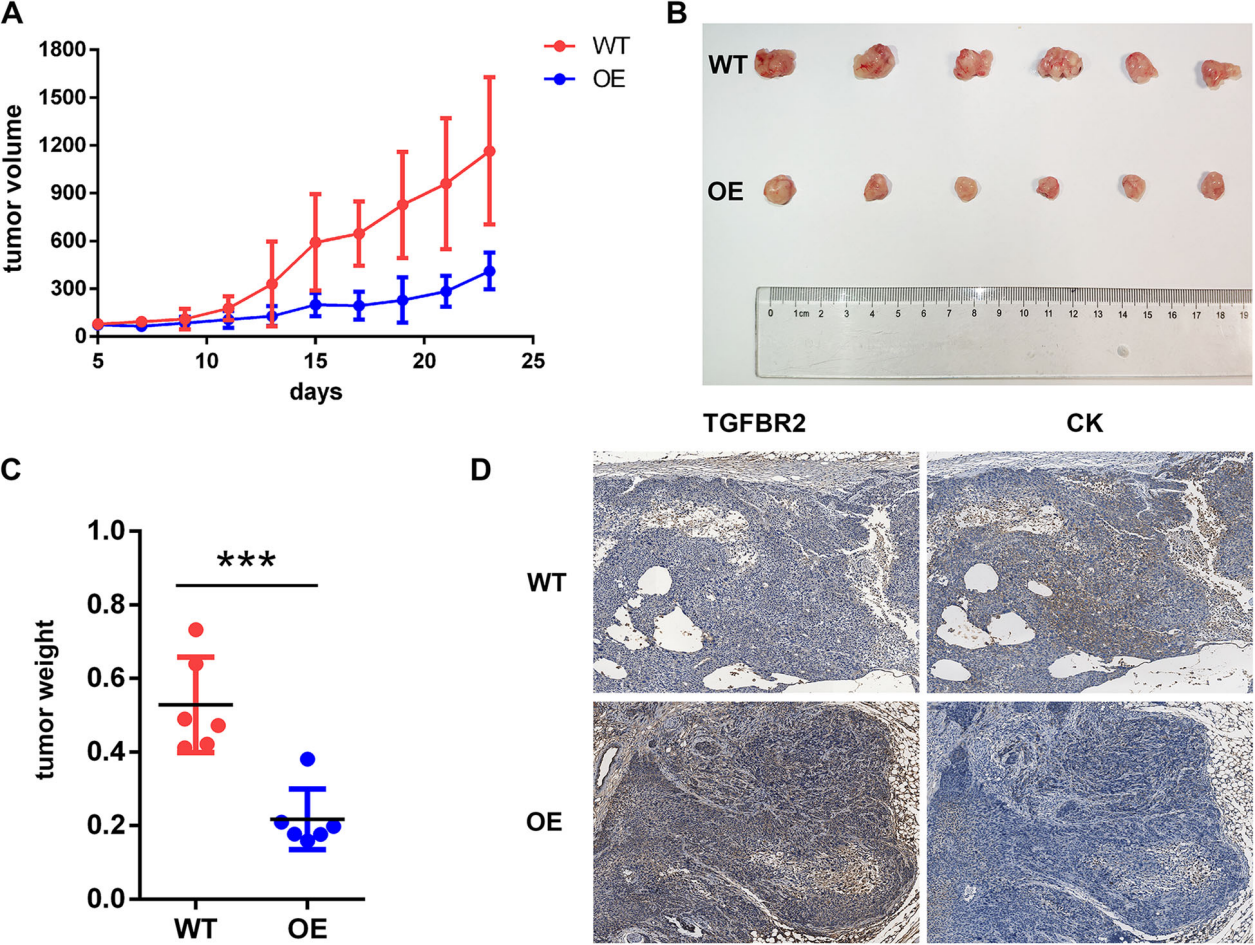
TGFBR2 overexpression inhibits tumor growth in vivo. **a** Tumor volume as measured in xenografts over 21 days derived from KYSE-150-vector (WT) and KYSE-150-TGFBR2 (OE) cells. **b** Image of subcutaneous xenografts derived from the cells indicated after growth in vivo for 3 weeks. **c** Tumor weight associated with WT- and OE-derived xenografts at 21 days. **d** IHC reveals the expression of TGFBR2 and CK in WT and OE cell xenografts

To determine whether TGFBR2 suppressed ESCC proliferation in vivo, we established a subcutaneous ESCC xenograft model in nude mice using KYSE-150-TGFBR2 and control KYSE-150-vector cells. Tumor weight and volume were decreased significantly in KYSE-150-TGFBR2 xenografts compared to KYSE150-vector xenografts (weight 0.22 ± 0.08 g and 0.53 ± 0.13 g, KYSE-150-TGFBR2 vs KYSE-150-vector; Fig. 7a–c). IHC confirmed that the level of TGFBR2 protein was upregulated and Pan-Keratin (CK) was downregulated in KYSE-150-TGFBR2 tumors when compared to the controls. There was a significantly negative correlation between TGFBR2 and CK protein which suggested that the overexpression of TGFBR2 can inhibit the proliferation of ESCC (Fig. 7d). Taken together, these results indicated that TGFBR2 expression significantly inhibited ESCC growth in vivo.

## Discussion

In the present study, we analyzed DNA methylation in different stages of ESCC carcinogenesis at single-base resolution. This approach allowed us to identify the methylation profiling in each ESCC patient and the exact boundaries of DMRs on a genome-wide scale. To the best of our knowledge, this is the first time methylation changes in ESCC have been characterized compared with those occurring in both normal esophageal and dysplasia tissues. In addition to the well-described genetic heterogeneity existing between dysplasia and ESCCs, we also observed epigenetic heterogeneity in each ESCC patient.

Carcinogenesis has been recognized as a composition of altered epigenetic and genetic events. Many cancers display global hypomethylation and site-specific hypermethylation at the CpG islands [22, 23]. We observed a similar genome-wide trend toward hypomethylation in the development of normal esophageal tissue into cancer. We also found that most DMRs were located in transcribed regions of the genome. However, we did not observe previously reported gene methylation changes, such as *CDKN2A* [18] and *TFF1* [14], in ESCC possibly due to our small sample size. Nevertheless, the hypermethylation pattern was identified to play an unequivocal character in the management of *TGFBR2* expression in ESCC. We further validated our bisulfite sequencing results through analysis of the TCGA ESCC dataset. Tumor samples showed higher methylation levels compared to normal esophageal tissue samples at *TGFBR2* promoter-related CpG sites. *TGFBR2* methylation was inversely correlated to its expression in tumor samples. In addition, *TGFBR2* promoter methylation was increased in tumors with copy number loss compared to those remaining intact at the gene site.

TGFBR2, as a member of TGF-β/Smad signal pathway, is an important tumor suppressor, which mediates TGF-β signaling and induces cell cycle arrest and apoptosis [24]. Under physiological condition, *TGFBR2* is normally expressed in the epithelial cells of esophageal mucosal. However, in pathological conditions, its expression specifically disappears [25, 26]. Downregulation or missense mutation of *TGFBR2* has been found in several cancers [27–29]. A previous study in vivo demonstrated that mice lacking *TGFBR2* expression developed anal or genital carcinoma. These results have indicated that loss of *TGFBR2* expression promotes epithelial oncogenesis. Several studies have shown that TGFBR2 inhibits cell growth, invasion, migration, and metastasis in breast and pancreatic cancer [30, 31]. TCGA consortium and others have reported the mutations of *TGFBR2* in ESCC [26]. Here, we provide evidences to favor an alternative hypothesis which DNA promoter methylation is a crucial mechanism leading to suppression of *TGFBR2* expression in ESCC. Furthermore, we identified specific regions methylated in the *TGFBR2* gene. Our study confirmed that *TGFBR2* expression was highly suppressed in ESCC cells and tumor tissues.

Hypermethylation of the CpG islands in promoter region is highly associated with silenced tumor-related genes through the reduction of mRNA transcription. Furthermore, gene expression could be rehabilitated with methylation inhibitors since DNA methylation is a reversible procedure. Thus, the regular growth regulation mode could be restored with demethylating genes before other genetic changes. In the present study, *TGFBR2* expression in ESCC cell lines could be restored with the demethylating reagent 5-Aza-2′-deoxycytidine. In addition, epigenetic silencing genes are often involved in several circuit of carcinogenesis, such as apoptosis, cell cycle, and DNA repair. The imbalance between cell growth and cell death can be recognized as an early and significant event in the carcinogenic process. Our results demonstrated that the overexpression of TGFBR2 or treatment with the demethylating agent 5-Aza-2′-deoxycytidine significantly induced cell cycle arrest in ESCC cell lines. Moreover, the overexpression of TGFBR2 suppressed ESCC growth in vivo. All together, we hypothesize that *TGFBR2* plays a role in suppressing ESCC tumorigenesis.

DNA methylation changes in tumor-related genes are frequent and early events during carcinogenesis [32]. Several methylation shifts occur during development from dysplasia to tumor. Methylation of specific sites may therefore be of biological and further clinical value in the early detection of ESCC, which is urgent for more favorable outcomes in the treatment of patients. Hotspots for DNA methylation are also valuable as biomarkers in the so-called liquid biopsy for cancer diagnosis and therapy since they are not only detected in resected tissues, but also in various body fluids, including peripheral blood [33–36], saliva [37–39], and urine [40–42]. In fact, methylated *APC* [43] and *CDKN2A* [44] have already been detected in the plasma of a subset of ESCC patients. The feasibility of the detection of *TGFBR2* methylation in serum of ESCC patients is therefore warranted.

## Conclusions

In summary, *TGFBR2* is downregulated in ESCC due to DNA hypermethylation of its promoter regions. The high level of methylated CpGs in *TGFBR2* in ESCC suggests that DNA methylation in *TGFBR2* promoter region would contribute to absent or reduced *TGFBR2* mRNA expression, and hence promote ESCC carcinogenesis. Cancer cells with treatment of DNA methyltransferase inhibitor 5-Aza-2′-deoxycytidine reversed methylation levels in the *TGFBR2* promoter and induced cell cycle arrest. Characterizing the role of *TGFBR2* in ESCC could pave the way to a deeper understanding of the potential mechanisms underlying disease development as well as illuminate its potential as a biomarker for early diagnosis and personalized therapeutic agent for ESCC patients.

## Methods

### Patient samples

Primary tissue samples were collected from patients who had undergone endoscopic surveillance and were analyzed by experienced pathologists. Individuals with ESCC were all inpatients who underwent surgical operations in Chinese PLA General Hospital between 2017 and 2018. Tissues were separated into two sections, one of which was stored at − 80 °C and the other was formalin fixed and paraffin embedded. Histological tumor characterization of resected specimens was carried out in the Department of Pathology in Chinese PLA General Hospital. All sample sections were stained in hematoxylin and eosin and were reviewed by two experienced pathologists. The pathology of the samples collected was the following: dysplasia, *n* = 6; and ESCC cancer, *n* = 3.

### Whole-genome bisulfite sequencing library preparation

DNA was extracted with DNeasy Blood and Tissue Kit (Qiagen; Valencia, CA, USA), and each DNA sample was spiked with 1% unmethylated lambda DNA (Promega; Madison, WI, USA) to evaluate the bisulfite conversion efficiency. The genomic DNA (500 ng) was fragmented with Covaris M220 ultrasonicator (Covaris; Woburn, MA, USA) to an average size of 350 bp. End repair and methylated adaptor ligation was conducted with NEBNext Ultra End Repair/dA-Tailing Module, Ligation Module and NEBNext Multiplex Oligos for Illumina (Methylated Adaptor, Index Primers Set 1; New England Biolabs; Ipswich, MA, USA). DNA fragments between 400 and 500 bp were selected for library construction with Ampure XP beads (Beckman Coulter; Brea, CA, USA). Bisulfite conversion was performed on samples using the EZ DNA Methylation kit (Zymo Research; Irvine, CA, USA) with modified single-stranded DNA fragments amplified using the Kapa HiFi U+ HotStart ReadyMix (Kapa Biosystems; Wilmington, MA, USA) with primers (NEBNext Multiplex Oligos for Illumina). A final size selection was performed to enrich the library for a range between 300 and 500 bp. Constructed libraries were evaluated on the Agilent 2100 Bioanalyzer (Agilent Technologies; Santa Clara, CA, USA) and then sequenced on the Illumina HiSeq X Ten (Illumina; San Diego, CA, USA) using the 150-bp paired-end mode.

### Whole-genome bisulfite sequencing data analysis

Sequencing reads were processed with the Bsmooth software package (http://rafalab.jhsph.edu/bsmooth), as described previously (PMID: 23034175). Briefly, reads were aligned to the human genome (hg19) together with the lambda phage genome using Bowtie2 v. 2.2.3. Following alignment, methylation measurements for each CpG site were obtained, and the bisulfite conversion rates were calculated based on the spiked-in unmethylated lambda phage DNA. To identify DMRs, the bsseq package in Bsmooth was utilized to smooth the data with default parameters (ns = 70, *h* = 1000), to characterize DMRs containing either 70 CpGs or a width of 1 kb, whichever was larger. Regions fulfilling the following criteria were deemed putative DMRs: (1) *t*-statistics passed the cutoff criteria of (− 3, 3); (2) containing at least three CpG sites; and (3) methylation difference of at least 10%. DMRs were then annotated with ANNOVAR software. Promoter regions of genes were defined as up to 1500 nt regions upstream of transcriptional start sites.

### Analysis of TCGA data

RNA-seq level 3 data, DNA methylation array data, mean segments of copy number, and clinical data of patients were downloaded from The Cancer Genome Atlas (TCGA) portal (https://portal.gdc.cancer.gov/). We chose FPKM values to represent gene expression levels (81 ESCCs and 11 normal tissue samples). FPKM values were then transformed into TPM values (transcript per million) to compare expression between samples. The methylation levels of CpG sites were measured using the Illumina Infinium Human Methylation 450 BeadArray platform and represented as the *β* value (81 ESCCs and 16 normal tissue samples; *β* value = intensity of the methylated allele/(intensity of the methylated allele + the unmethylated allele). Differentially methylated or expressed analyses were performed using the Mann-Whitney *U* test. The Benjamini-Hochberg method was applied for adjusting *P* values to control the false discovery rate. The CpG sites with adjusted *P* values less than 0.05 were considered to be differentially methylated. For copy number variation analysis, a segment mean of 0.5 was defined as the cutoff for amplifications and − 0.5 for deletions. Survival analysis was conducted with the Kaplan-Meier method, and the log-rank test was performed to test difference in survival between two groups. Optimal cutoff value for *TGFBR2* expression in survival analysis was determined using maximally selected rank statistics.

### Cell lines and cell culture

The cell lines KYSE-150 and KYSE-30 were kindly gifted from Dr. Shimada Y (Kyoto University, Kyoto, Japan). All ESCC cell lines were cultured in RPMI1640 medium supplemented with 10% FBS. The cell line Het-1A was acquired from ATCC and cultured in BEGM™ medium prepared with the Bronchial Epithelial Cell Growth Medium Bullet Kit along with all the additives (Lonza/Clonetics Corporation, CC-3170; Hayward, CA, USA).

### Construction of stable TGFBR2 expressing cell lines

To generate lentivirus, the *TGFBR2* lentiviral plasmid (pLVX-IRE-Puro-TGFBR2; Wuhan Miaoling Bioscience & Technology Co., Ltd; Wuhan, China) was cotransfected with the psPAX2 and pMD2.G plasmids using Neofect™ DNA transfection reagent (1 μL/mL; Neofect; Beijing, China) for packaging in HEK-293 T cells. The viruses were harvested 72 h after transfection. KYSE-150 cells were transduced with the lentivirus. Stably infected cells were selected in puromycin (2 μg/mL; Life Technologies, Waltham, MA, USA) for 2 days and confirmed by RT-qPCR and western blot.

### Quantitative real-time PCR

Total RNA was extracted from cultured cell lines with TRIzol (Thermo Fisher Scientific; Waltham, MA, USA), and cDNA was synthesized with the PrimeScript™ RT Master Mix (TaKaRa; Beijing, China). Quantitative real-time PCR was performed in triplicate using TB Green™ Premix Ex Taq™ (Tli RnaseH Plus; TaKaRa) on the ABI (7900HT) system (Applied Biosystems; Foster City, CA, USA). The expression of *TGFBR2* was calculated using the 2^−△△CT^ method. The primer sequences used are the following: *TGFBR2* forward primer: 5′-GTAGCTCTGATGAGTGCAATGAC-3′; *TGFBR2* reverse primer: 5′-CAGATATGGCAACTCCCAGTG-3′; *GAPDH* forward primer: 5′-GGAGCGAGATCCCTCCAAAAT-3′; *GAPDH* reverse primer: 5′-GGCTGTTGTCATACTTCTCATGG-3′.

### Western blot analysis

Cells were harvested and lysed in RIPA lysis buffer. Protein concentrations were evaluated by the BCA protein assay kit (PLYGEN, China) according to the manufacturer’s instructions. The protein lysates were separated on 10% SDS-PAGE and electrophoretically transferred to polyvinylidene fluoride (PVDF) membranes. The membranes were overnight incubated with primary antibodies at 4 °C. The protein bands were detected and quantitated using enhanced chemiluminescence (ECL).

### Colony formation assay

For colony formation assay, cells were seeded onto 6-well plates with 800 cells per well and the medium was changed every 3 days. After 10 days, cells were fixed with 4% formaldehyde for 20 min and stained with 1% crystal violet solution for 10 min.

### Flow cytometry

KYSE-150 cells were incubated with different dosages of DNA methyltransferase inhibitor for 48 h before flow cytometry analysis. DNA methyltransferase inhibitor 5-Aza-2′-deoxycytidine was acquired from MedChemExpress (Monmouth Junction, NJ, USA) and fully dissolved in dimethylsufoxide (DMSO) at a concentration of 10 mM. Apoptosis was assessed using the Annexin V, 633 Apoptosis Detection Kit (Dojindo, Kumamoto, Japan). Cell cycle analysis was performed using the Cell Cycle and Apoptosis Analysis Kit (Beyotime; Jiangsu, China). Both assays were carried out and analyzed on a flow cytometer (Beckman Coulter) according to the manufacturer’s instructions. Data from the apoptosis assay were analyzed using FlowJo v10 (FlowJo, LLC). Cell cycle distributions were statistically determined by Modfit LT 3.2 software (Verity Software House; www.vsh.com; Topsham, ME).

### Immunohistochemistry (IHC)

Immunohistochemistry was performed using an indirect peroxidase method. Paraffin embedded sections of esophageal tissues on slides were dewaxed fully in xylene and rehydrated thoroughly in a decreasing graded series of ethanol concentrations. Endogenous peroxidase was quenched with 3% hydrogen peroxide, and sections were blocked with 10% goat serum (ZSGB-BIO; Beijing, China) to reduce non-specific binding of antibodies. All tissues were incubated overnight with primary antibody at 4 °C. Antibody against TGFBR2 was obtained from Abcam (Shanghai, China). For detection, slides were returned to room temperature and incubated with horse radish peroxidase (HRP)-labeled goat anti-rabbit IgG (1:200, Proteintech, Wuhan, China). Diaminobenzidine (DAB, ZSGB-BIO, Beijing, China) was used as the chromogenic substrate. Slides were counterstained with hematoxylin and mounted in resin. Images were acquired through Aperio pathology scanner.

### Xenografts

KYSE-150 cells and KYSE-150-TGFBR2-overexpressed cell suspensions were subcutaneously injected into female BALB/c nude mice (age 4–5 weeks; *n* = 6 in each group). Tumor volumes were subsequently measured every 3 days and calculated (volume = *R* × *r*^2^/2, *R* represents the longest diameter and *r* represents the shortest diameter). After 3 weeks, the mice were sacrificed, and tumor samples were processed for further analysis.

## Supplementary information

**Additional file 1: Figure S1**. TGFBR2 mRNA levels in several ESCC cell lines. (**A**) RT-qPCR to detect levels of TGFBR2 mRNA in Het-1A and ESCC cell lines using ACTB as a control gene. (**B**) RT-qPCR to detect levels of TGFBR2 mRNA in Het-1A and ESCC cell lines using 18S RNA as a control gene.

## Data Availability

All the datasets are available from the corresponding author upon reasonable request.

## Abbreviations

DAB: Diaminobenzidine
DMRs: Differentially methylated regions
DMSO: Dimethylsufoxide
EC: Esophageal cancer
ECL: Enhanced chemiluminescence
ESCC: Esophageal squamous cell carcinoma
HRP: Horse radish peroxidase
IHC: Immunohistochemistry
MeDIP-Seq: Methylated DNA immunoprecipitation sequencing
MSP: Methylation-specific PCR
PVDF: Polyvinylidene fluoride
TCGA: The Cancer Genome Atlas
TGFBR2: Transforming growth factor-β receptor type II gene
TGF-β: Transforming growth factor-β
WGBS: Whole genome bisulfite sequencing
