# Supplementary figures and images for: Methylation silencing of TGF-β receptor type II is involved in malignant transformation of esophageal squamous cell carcinoma
Source: Clin Epigenetics. 2020 Feb 11;12:25. doi: 10.1186/s13148-020-0819-6 (PMC7014638; doi:10.1186/s13148-020-0819-6)

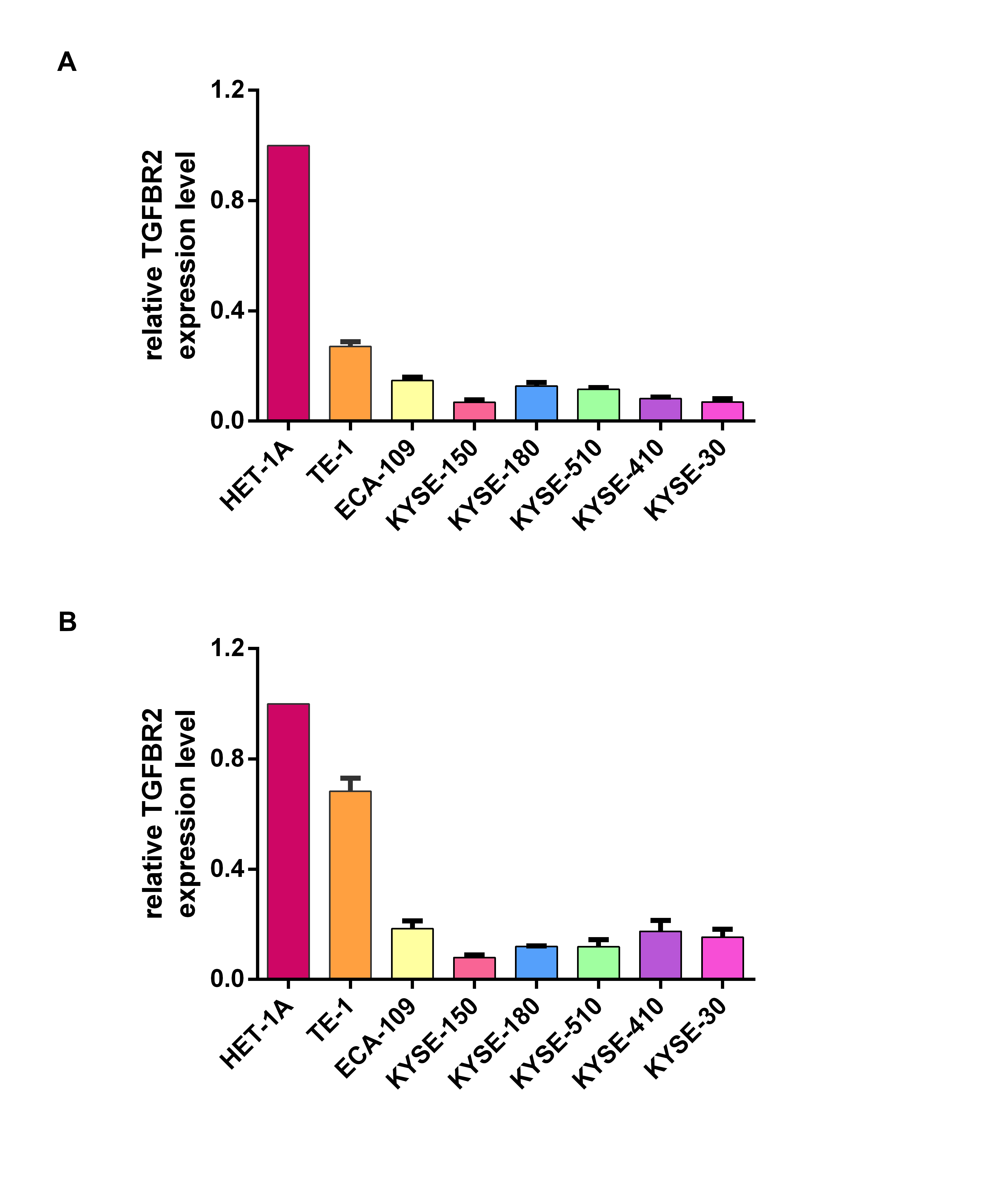

Supplement: Supplementary file 1 — Additional file 1: Figure S1. TGFBR2 mRNA levels in several ESCC cell lines. (A) RT-qPCR to detect levels of TGFBR2 mRNA in Het-1A and ESCC cell lines using ACTB as a control gene. (B) RT-qPCR to detect levels of TGFBR2 mRNA in Het-1A and ESCC cell lines using 18S RNA as a control gene. [file 13148_2020_819_MOESM1_ESM.tif]
